# Aggregation States of A*β*_1–40_, A*β*_1–42_ and A*β*p_3–42_ Amyloid Beta Peptides: A SANS Study

**DOI:** 10.3390/ijms20174126

**Published:** 2019-08-24

**Authors:** Giulia Festa, Francesco Mallamace, Giulia Maria Sancesario, Carmelo Corsaro, Domenico Mallamace, Enza Fazio, Laura Arcidiacono, Victoria Garcia Sakai, Roberto Senesi, Enrico Preziosi, Giuseppe Sancesario, Carla Andreani

**Affiliations:** 1 CENTRO FERMI—Museo Storico della Fisica e Centro Studi e Ricerche “Enrico Fermi”, 00184 Rome, Italy; 2 Department of Nuclear Science and Engineering, Massachusetts Institute of Technology, Cambridge, MA 02139, USA; 3 IRCCS Fondazione Santa Lucia, 00142 Rome, Italy; 4 Department of Experimental Medicine and Surgery, Università degli Studi di Roma “Tor Vergata”, 00133 Rome, Italy; 5 Dipartimento di Scienze Matematiche e Informatiche, Scienze Fisiche e Scienze della Terra (MIFT), Università di Messina, 98166 Messina, Italy; 6 Science and Technology Facilities Council, ISIS Pulsed Neutron and Muon Source, Didcot OX11 0QX, UK; 7 NAST Centre and Department of Physics, Università degli Studi di Roma “Tor Vergata”, 00133 Rome, Italy; 8 Department of Systems Medicine, Università degli Studi di Roma “Tor Vergata”, 00133 Rome, Italy

**Keywords:** beta amyloid, aggregation state, small angle neutron scattering, Alzheimer’s disease

## Abstract

Aggregation states of amyloid beta peptides for amyloid beta Aβ${}_{1–40}$ to Aβ${}_{1–42}$ and Aβp${}_{3–42}$ are investigated through small angle neutron scattering (SANS). The knowledge of these small peptides and their aggregation state are of key importance for the comprehension of neurodegenerative diseases (e.g., Alzheimer’s disease). The SANS technique allows to study the size and fractal nature of the monomers, oligomers and fibrils of the three different peptides. Results show that all the investigated peptides have monomers with a radius of gyration of the order of 10 Å, while the oligomers and fibrils display differences in size and aggregation ability, with Aβp${}_{3–42}$ showing larger oligomers. These properties are strictly related to the toxicity of the corresponding amyloid peptide and indeed to the development of the associated disease.

## 1. Introduction

The formation of neuritic plaques throughout the grey matter of the brain, associated with neurofibrillary tangles and neuronal loss, is responsible for neurodegenerative illnesses, such as the Alzheimer’s disease (AD) [1]. Electron microscopy studies have revealed that the plaques comprise a central mass of extracellular filaments called amyloid fibrils that radially extend toward the periphery, where they are tangled with dystrophic neurites as well as astrocytic and microglial processes [1]. Unlike extracellular amyloid fibrils, the neurofibrillary tangles are fibril aggregates of the microtubule-associated protein tau, which has become hyperphosphorylated and accumulates inside the cells themselves [1].

Clinicopathological correlation studies have established that the neuritic plaque build-up occurs primarily in the brain before the onset of cognitive deficits, while neurofibrillary tangles and neuronal and synaptic loss are directly related to cognitive decline [1,2].

While many questions about the importance of plaques in the pathogenesis of AD remain unsolved, the abnormal accumulation of neuritic plaques in various areas of the brain in AD precedes and, with time, is invariably associated with neurodegeneration [2,3]. While the frequency and topographical extension of neuritic plaques increase with disease progression [4,5], their size inexplicably does not significantly grow over an average 50 μm in diameter [6,7].

Plaques contain amyloid fibrils made up of aggregates of small peptides, of 39–43 amino acids in length, called amyloid beta (Aβ), resulting from the sequential proteolytic cleavage of the amyloid precursor protein (APP) by the enzymes β- and γ-secretases [8]. Among Aβ polypeptides, A$\beta_{1–42}$ shows the highest aggregation propensity, and its deposition precedes the deposition of Aβ${}_{1–40}$ in vivo [9]. In addition, N-truncated and pyroglutamate (pGlu)-modified Aβ peptides, e.g., Aβ${}_{5–40/42}$, A$\beta_{3–40/42}$ and Aβp${}_{5–40/42}$, are major constituents of amyloid deposits in sporadic and inherited AD and show an enhanced aggregation propensity compared to full-length peptides [9]. pGlu-modified peptides are prominent in AD and have been postulated to initiate amyloid plaque formation [9,10]. However, Bayer et al. [11] hypothesized that N-truncated Aβ peptides form, preferentially, higher molecular weight soluble toxic oligomers which have a reduced tendency to aggregate in plaques [11].

Despite the enormous advances of the knowledge on the biochemical composition of plaques, the dynamics of the plaque deposition in vivo throughout the cerebral cortex, the “stop and go” factors leading to the formation of innumerable plaques of limited size, remain largely unknown. In fact, the ability of different Aβ polypeptides to aggregate into soluble oligomers and form insoluble protofibrils and fibrils can be explored only in vitro. Several experimental techniques are available to measure protein aggregation, but only a few can measure kinetics of the early stage of aggregation from monomers to small oligomers and fibril formation in aqueous solvents [12]. Recently, the small angle neutron scattering (SANS) technique was shown to be very useful to study aggregates and aggregation kinetics of small peptides like Aβ [13].

In the present study, SANS measurements are carried out to compare the aggregation state and the size of aggregates of monomers, oligomers and fibrils of some peptides involved in neurodegenerative pathologies, such as Alzheimer’s disease and Parkinson’s disease with dementia [14]. The aim of this work is extending the analysis made in [14] on Aβ${}_{1–40}$ to other peptides such as Aβ${}_{1–42}$ and Aβp${}_{3–42}$, using the first as a basis for comparison. For this reason, in the following analyses it will be specified when data and results from [14] have been used.

## 2. Results and Discussion

### 2.1. Formation of Synthetic A$\beta_{1–40}$, A$\beta_{1–42}$ and A$\beta p_{3–42}$ Aggregates

The in vitro presence of monomers, and formation of aggregates of the different amyloid β peptides, was confirmed by running all samples in SDS-PAGE, followed by silver staining. However, representative SDS-PAGE has been used just to verify the presence of monomer, oligomer and fibrillary aggregates; the silver staining analysis is not intended as a definitive identification of all the various aggregation species prepared in vitro [15] and finally observed by SANS.

**Figure 1:**
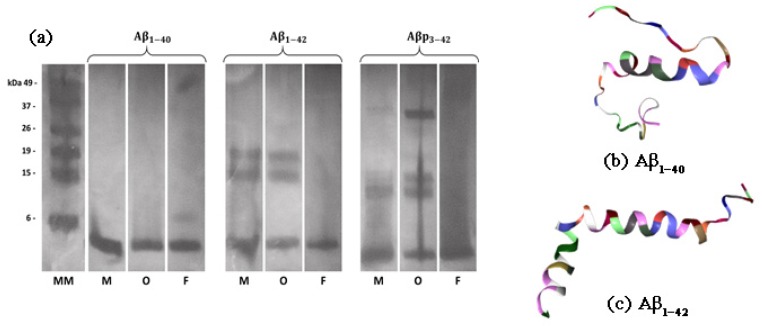
(**a**) Image from SDS-PAGE analysis. Silver stain of synthetic aggregates and monomers of A$\beta_{1–40}$, A$\beta_{1–42}$ and A$\beta p_{3–42}$ peptides. In monomeric preparations the peptides show a band at ≃4 kDa; A$\beta_{1–42}$ and Aβp${}_{3–42}$ form spontaneous aggregates of low molecular weight (in the 11–19 kDa range). Synthetic A$\beta_{1–42}$ and Aβp${}_{3–42}$ show bands of low and high molecular weight in aggregating conditions, while synthetic A$\beta_{1–40}$ did not form oligomers and fibrils in the same conditions. The letters under the images describe the aggregation state of the samples (M = monomers, O = oligomers, F = fibrils) while the leftmost image is the molecular marker reference. The structure of (**b**) A$\beta_{1–40}$ (PDB ID 2LFM) and (**c**) A$\beta_{1–42}$ (PDB ID 1IYT) is shown on the right side.

In monomeric preparations, A$\beta_{1–40}$, A$\beta_{1–42}$ and Aβp${}_{3–42}$ peptides show a band at about 4 kDa; however, A$\beta_{1–42}$ and Aβp${}_{3–42}$ monomeric preparations also show the presence of spontaneous aggregates of low molecular weight (Figure 1). As expected, in oligomeric conditions, A$\beta_{1–42}$ and Aβp${}_{3–42}$ show bands of low molecular weight in addition to the band at 4 kDa corresponding to the monomers; moreover, Aβp${}_{3–42}$ shows also high molar weight (∼37 kDa) oligomers. In contrast, in oligomeric conditions A$\beta_{1–40}$ does not form SDS stable oligomers [15], showing only the monomer band in aggregating conditions. In fibrillar conditions A$\beta_{1–40}$, A$\beta_{1–42}$ and Aβp${}_{3–42}$ only show the monomer band, but not the low or high molecular weight oligomers, whereas fibrils are upstream in the gel column out of the reference molecular mass (column on the left in Figure 1).

### 2.2. SANS Results and Discussions of the G, $R_{g}$ and d Parameters

**Figure 2:**
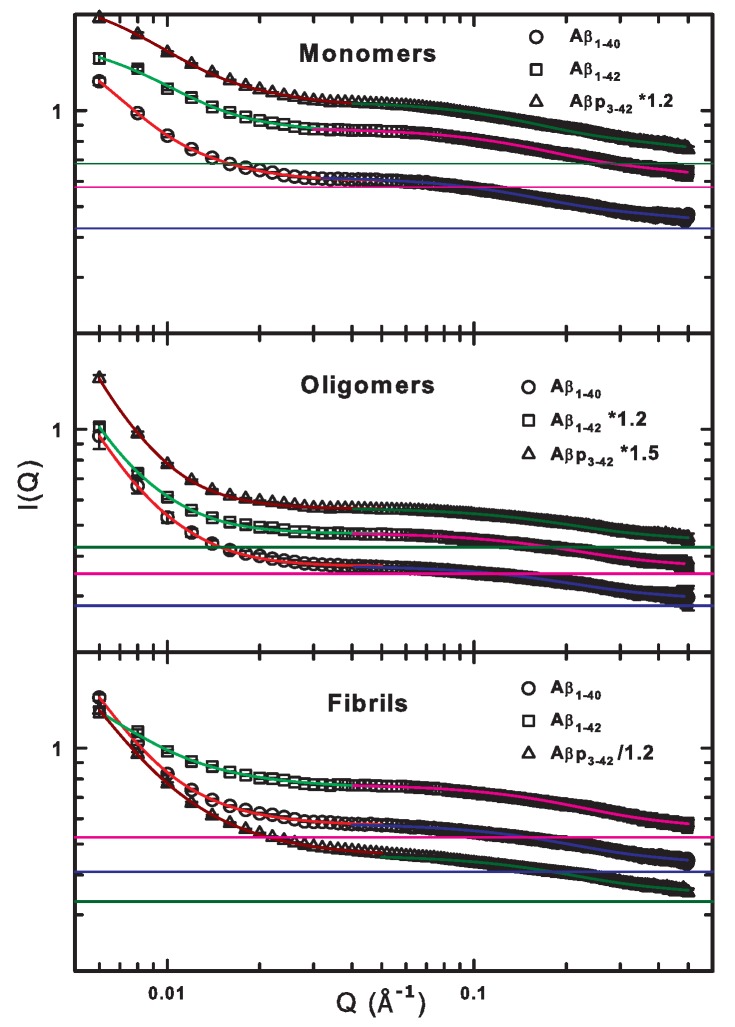
Best fit for small angle neutron scattering (SANS) spectra for monomers (top), oligomers (middle) and fibrils (bottom) of the A$\beta_{1–40}$, A$\beta_{1–42}$ and Aβp${}_{3–42}$ peptides. The data are fitted with the Beaucage model in two wave-vector regimes: At low Q values (Q < 0.045 Å${}^{-1}$) and at high Q values (0.045 Å${}^{-1}$ < Q < 0.5 Å${}^{-1}$), highlighted with different colors. The color horizontal lines represent the background value obtained from high Q data fit for each system. Some data have been scaled by a factor reported in the figure legend to avoid plots overlapping. Note that the experimental error bars are smaller than symbol size (except for a few points).

Figure 2 shows fits to the SANS data using the Beaucage model in two Q ranges (see the section “Materials and Methods”), with the background levels obtained from fits to high Q values. For comparison, the data from monomers, oligomers and fibrils for A$\beta_{1–40}$, previously reported in [14], are also shown. Not surprisingly, our analysis shows that, for monomers in the high Q region, the radius of gyration ($R_{g}$) has a size of about 10.6 Å for all the peptides (see Table 1), as expected from the literature [16]. For low Q values, $R_{g}$ ranges from 175 to 250 Å, consistent with an aggregate made up of many monomeric unities. The values of the exponent *d* are consistent with the assignment, ranging between 2.4 and 3 for low Q values, suggesting the presence of collapsed chains, while being close to 1 for high Q, according to an open chain conformation [17]. The Guinier scale factor *G* is about 1 cm${}^{-1}$ for low Q and ranges from 0.19 cm${}^{-1}$ to 0.32 cm${}^{-1}$, suggesting, in both cases, small scattering objects, a result coherent with a low degree of polymerization.

The oligomer data, shown in the middle panel of Figure 2, that fit to the high Q region give $R_{g}$ values of about 8.2 Å, smaller than those from monomers, which can be explained by the presence of a surface layer with higher density, as reported in [13]. For low Q values, $R_{g}$ has a size ranging from 380 Å to 540 Å (compatible with the size of medium-size oligomers [18]). The *d* exponent is about 3 at low Q and about 1 at high Q for all three peptides, similar to that observed for the monomers. In contrast, the Guinier scale factor for the low Q region is larger for oligomers than for monomers, suggesting a higher degree of aggregation. For the high Q region, the value is around 0.1 cm${}^{-1}$, like for the monomers.

The behavior of fibril samples in the high Q region is similar to that of the oligomers with the values $R_{g}$ and *d* being similar at about 8 Å and 1.3, respectively. The value of the parameter *G* (0.2 cm${}^{-1}$) is similar to that of monomers and slightly larger than that of the oligomers.

Therefore, within the fibril samples, monomeric species are present, although they show a dimension that is smaller than that within the monomeric solution. Again, this is compatible with the presence of a surface layer with higher density [13]. In contrast, in the low Q region, fibrils have much larger $R_{g}$ compared to monomers or oligomers (in some cases even five times larger), with A$\beta_{1–40}$ (706 ± 52 Å) being smaller than Aβp${}_{3–42}$ (771 ± 33 Å), than A$\beta_{1–42}$, which shows the highest value (880 ± 85 Å). The parameter *d* varies between 2 for Aβp${}_{3–42}$ and 2.4 for Aβ${}_{1–40}$, indicating the presence of collapsed chains with an increasing packing degree. Finally, *G* takes on the value of about 11 cm${}^{-1}$ for all the three peptides. This value is higher with respect to that of monomers and oligomers because of the presence of bigger scattering objects.

It should be noted that, although the uncertainties given in Table 1 are strictly related to the analysis performed with the Beaucage model, they provide information about the distribution width of the corresponding values. In addition, it is important to highlight that the obtained values also depend on the dispersity of the different species in solutions and that this factor contributes to their variability, too. Hence, a greater uncertainty in R${}_{g}$ means a bigger size distribution. This is the case, for example, for the oligomers and fibrils for the Aβ${}_{1–42}$ peptide. The model used to investigate the peptides allows differentiation on the basis of size, highlighting the co-existence of two different phases. The value of the parameters, especially at low Q, makes it possible to distinguish different kinds of aggregates in solution. For instance, it is noteworthy that our results show that the oligomers of Aβp${}_{3–42}$ are greater and show a higher degree of polymerization with respect to the oligomers from the other two peptides.

The SANS results presented here are consistent with findings from dynamic light scattering analysis [13] made on Aβ${}_{1–40}$ and Aβ${}_{1–42}$, where the formation of aggregates was monitored with time. That same study finds two relaxation processes, at 20 μs and 5000 μs, corresponding to objects with a hydrodynamic radius ranging from 20 Å to 5000 Å.

As previously mentioned, the signals for all the SANS measurements shown in Figure 2 are affected by a background. By subtracting this contribution from the signal and plotting the subtracted data versus Q multiplied by the hydrodynamic radius (Q · R${}_{H}$), it is possible to verify the data range in which the transition from the Guinier to the Porod regime (at Q · R${}_{H}$ = 1) takes place. Moreover, from the slope of the high Q · R${}_{H}$ data in a log–log plot, information about the presence of fractal aggregates can be inferred [19].

**Figure 3:**
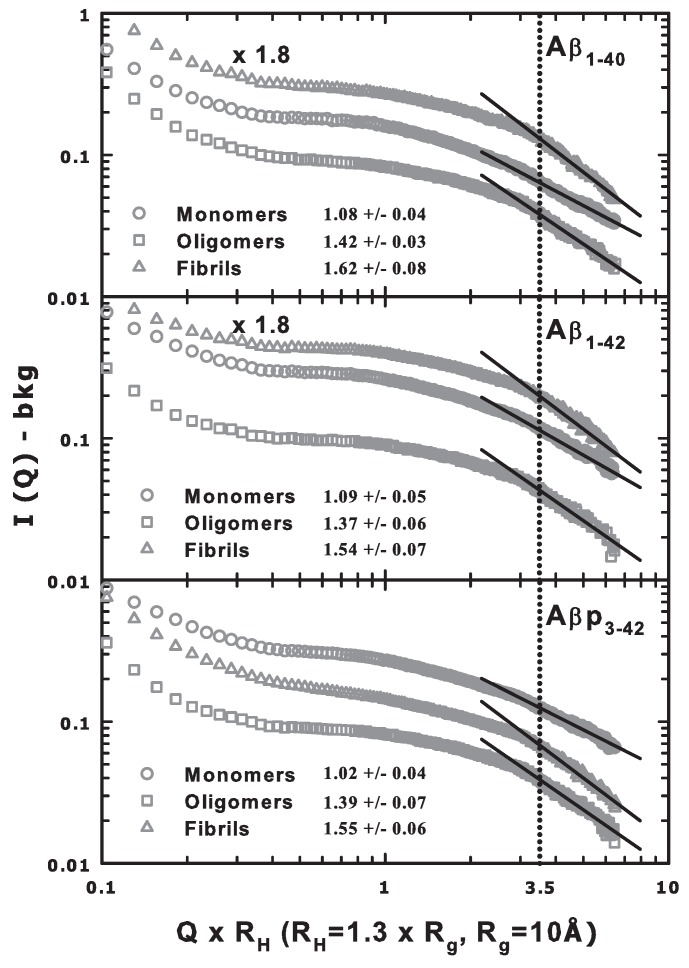
Background subtracted data as a function of Q · R${}_{H}$, with R${}_{H}$ = 13 Å, in a log–log plot. for monomers, oligomers and fibrils of the three investigated peptides. Data of fibrils in the top and middle panels have been rescaled by multiplying for 1.8 to avoid overlaps. We have performed a linear best-fit for Q · R${}_{H}\geq 3.5$ (Porod region) to get indications about the fractal structure of the systems and the obtained slopes are reported in the corresponding legend.

Figure 3 shows such plots for all the investigated amyloid samples, yielding average slopes of the linear fits in the high (Q · R${}_{H}$) region of about 1.06, 1.39 and 1.57 for monomers, oligomers and fibrils, respectively. The precise and specific values are reported in the corresponding figure legend. The obtained values suggest the presence of an open structure for the monomers, a more compact one for the oligomers and the so-called diffusion limited cluster–cluster aggregation (DLCCA) [19] for the fibrils. However, the small observed differences among the slopes can be ascribed to a slightly different aggregation ability of the different peptides [19,20].

It is worth mentioning that the signal from the oligomers is systematically lower than the ones from monomers and fibrils. This effect could be due to the choice of the solvent (HAMs F12) that promotes the aggregation but that also disappears during the lyophilization process, differently from the DMSO and HCl used for the monomer and fibril samples, respectively [14]. This could also be the cause of the bigger spread shown by oligomer data at the highest values of Q · R${}_{H}$, especially for A$\beta_{1–42}$ and Aβp${}_{3–42}$. Furthermore, it must be taken into account that one of the most important forces that drives aggregation phenomena is the hydrophobic interaction, which is in competition with the hydrophilic one [21]. Hence, the physical properties of the solvent as well as their temperature and concentration dependencies have great relevance in the development of Alzheimer’s disease [22].

## 3. Materials and Methods

### 3.1. Preparation of Aβ Aggregates and Their Analysis by SDS-PAGE

Synthetic A$\beta_{1–40}$, Aβ${}_{1–42}$ and Aβp${}_{3–42}$ peptides were purchased from Anaspec (Fremont, CA, USA) and American Peptide (Sunnyvale, CA, USA). As previously reported in [15], the aggregates of peptides were obtained via the Dahlgren’s [23] modification of Lambert’s protocol [24]. Firstly, 6 mg of each peptide was dissolved to 1 mM in 100% hexafluoroisopropanol (HFIP; catalog number H8508; Sigma-Aldrich S.r.l., Milan, Italy) and sonicated for about 20 min, to disrupt eventual previous amorphous aggregates; then HFIP was removed under vacuum, and the peptides (Aβ${}_{1–40}$, Aβ${}_{1–42}$ and Aβp${}_{3–42}$ monomers) were dissolved in anhydrous dimethyl sulfoxide (DMSO bought from Sigma-Aldrich S.r.l., Milan, Italy) to 5 mM and sonicated for 10 min. At this point, in order to obtain oligomers or fibrils, two different incubation protocols were followed. For the oligomers, the monomeric solutions were diluted to 2 mg/mL in cold HAM’s-F12 culture medium (Euroclone) and kept for about 12 h at 4 ${}^{\circ }$C. For fibril formation, the samples were diluted in HCl 10 mM and incubated at 37 ${}^{\circ }$C for about 12 h. For un-aggregated conditions, the 5 mM Aβ${}_{1–40}$, Aβ${}_{1–42}$ and Aβp${}_{3–42}$ monomers in Me${}_{2}$SO were diluted directly into H${}_{2}$O. All samples were lyophilized and stored at –20 ${}^{\circ }$C. The samples were finally thawed at room temperature and resuspended in 0.5 mL of deuterium oxide (D${}_{2}$O) at concentrations of 2 mg/mL before the neutron study.

### 3.2. Preparation of Aβ for SANS2D

Samples of Aβ${}_{1–40}$, Aβ${}_{1–42}$ and Aβp${}_{3–42}$ monomers, oligomers and fibrils were prepared with a concentration of 2 mg/mL and filled into 2 mm path length standard SANS ‘tank’ cuvettes for the largest beam size possible with the aim to improve the signal-to-background ratio. All measurements were made at 37 ${}^{\circ }$C to simulate physiological conditions. Measurements were taken on 3 samples of every peptide and each aggregation state. The duration of a single measurement was 2 h per measurement and 3 repetitions were made to improve the statistics and to check for any changes as a function of time. The three repeats refer to runs registered at 2, 4 and 6 h from the solution production. It was important to keep the total measurement time below 10 h, which is the time of confirmed stability at physiological temperatures. In addition to these, 3 solvent samples of the peptide media in each case (DMSO/F12 for monomers and oligomers; HCl for fibrils) were also measured for subtraction purposes during the data analysis.

### 3.3. SDS-PAGE Analysis

Preliminary analyses concerning the structure of the aggregates were executed through sodium dodecyl sulfate–poly-acrylamide gel electrophoresis (SDS-PAGE) [25]. The formation of Aβ aggregates was assessed through SDS-PAGE analysis without heat denaturation and followed by silver staining. The monomers and the aggregated forms of the peptides (about 1μg of monomers for each sample) were run in 16% SDS-PAGE tris-tricine buffer and, subsequently, the gels were fixed in 40% methanol and 10% acetic acid for about 12 h and then were silver stained (with the Bio-Rad Silver Stain kit).

### 3.4. The Small Angle Neutron Scattering Technique

SANS uses elastic neutron scattering at small scattering angles to investigate the structure of materials at a mesoscopic scale. Due to the neutron–matter interaction, SANS has high sensitivity to light elements and the scattering pattern is analyzed to provide information about the size, shape and orientation of some components of the sample [26]. The measurements were carried out on the SANS2D instrument at ISIS pulsed neutron and muon source [14,27]. Samples were measured in circular quartz cuvettes at 37 ${}^{\circ }$C. The incident beam was collimated at 12 mm with the front and rear detector at 4 and 2.4 m from the sample, respectively. This configuration allowed for a Q range of 0.005 Å${}^{-1}$< Q < 1.5 Å${}^{-1}$ to be covered in a single measurement. Data were corrected and reduced to an absolute scale using a polymer standard [28].

Since no significant differences were found for different runs belonging to the same sample (the repeats), the data were merged to increase measurement statistics, confirming at the same time the stability of the samples diluted in D${}_{2}$O and the safety of the neutron–matter interaction within the duration of the experiment (<10 h).

### 3.5. SANS Analysis

The SANS data were treated and analysed following the same protocol as used for the analysis of data for the Aβ${}_{1-40}$ peptide, as described in our previous work [14]. The analysis is based on the Beaucage model [17,18] and gives information such as the Guinier scale factor (*G*), the Porod scale factor (*C*), the Porod exponent (*d*) and the radius of gyration ($R_{g}$). The latter represents an estimation of the particle size and is related to the hydrodynamic radius ($R_{H}$) by $R_{g}$ = 0.774 $R_{H}$. We used the model and the data analysis was performed in two different wave vector regions (Q < 0.045 Å${}^{-1}$ and 0.045 Å${}^{-1}<$ Q < 0.5 Å${}^{-1}$) because, as suggested by the SDS-PAGE analysis, the system consists of two phases. More specifically, the basic unit (i.e., the monomer) is always present (and detectable also in the high Q region) together with the corresponding aggregate state of dimensions gradually increasing from oligomers to fibrils. By means of the Beaucage model, it is possible to infer the shape and size of the proteins in the different aggregation states from the corresponding values of the *G*, $R_{g}$ and *d* parameters.

## 4. Conclusions

The data presented here extend the study of the aggregation states of amyloid beta peptides (begun with the work reported in [14] for Aβ${}_{1–40}$) to Aβ${}_{1–42}$ and Aβp${}_{3–42}$ using SANS. Since the aggregates are responsible for neurodegenerative diseases (e.g., Alzheimer’s disease), it is important to understand their structure and aggregation states. The SANS technique has shown a great capability in discriminating the size and fractal nature of the monomers, oligomers and fibrils of the different peptides. For this reason, SANS turned out to be a promising and engaging technique for the study of peptides beyond the more conventional ones. Our data show that, although the monomers of the three investigated peptides show a similar radius of gyration ($R_{g}$) of about 10 Å, the oligomers and fibrils show quite different sizes and aggregation abilities. Our data support the Bayer et al. hypothesis that A$\beta p_{3–42}$ has a greater propensity to form low and intermediate molecular weight soluble toxic oligomers and reduced non-toxic fibrillization activity than A$\beta_{1–42}$, explaining the different toxicity of the two peptides [11]. This information is crucial for the determination of the toxicity of the corresponding amyloid peptide and can help to foresee the rate of the illness development.

## Figures and Tables

**Table 1:** Fit parameters obtained by using the Becauge model at low Q (Q < 0.045 Å${}^{-1}$) and at high Q (0.045 < Q < 0.5 Å${}^{-1}$).

		G (cm${}^{-1}$)	R${}_{g}$ (Å)	d	bkg (cm${}^{-1}$)
	**low Q**				
	Monomers	1.30 ± 0.07	253.3 ± 9.6	2.8 ± 0.2	0.596 ± 0.006
	Oligomers	2.50 ± 0.56	377.9 ± 40.6	3.1 ± 0.1	0.371 ± 0.001
	Fibrils	11.72 ± 1.34	705.6 ± 51.7	2.4 ± 0.1	0.570 ± 0.002
A$\beta_{1–40}$					
	**high Q**				
	Monomers	0.19 ± 0.01	10.9 ± 0.4	1.2 ± 0.2	0.427 ± 0.007
	Oligomers	0.09 ± 0.01	8.1 ± 1.0	1.3 ± 0.5	0.278 ± 0.012
	Fibrils	0.17 ± 0.03	8.4 ± 0.9	1.3 ± 0.6	0.409 ± 0.026
	**low Q**				
	Monomers	0.88 ± 0.03	174.8 ± 5.7	3.1 ± 0.4	0.857 ± 0.006
	Oligomers	2.61 ± 0.72	425.9 ± 45.1	3.1 ± 0.4	0.392 ± 0.002
	Fibrils	11.49 ± 3.22	879.6 ± 85.8	2.1 ± 0.1	0.753 ± 0.002
A$\beta_{1–42}$					
	**high Q**				
	Monomers	0.30 ± 0.01	10.1 ± 0.4	1.1 ± 0.2	0.575 ± 0.014
	Oligomers	0.10 ± 0.02	8.1 ± 1.0	1.3 ± 0.8	0.294 ± 0.019
	Fibrils	0.24 ± 0.06	7.8 ± 1.2	1.3 ± 0.8	0.525 ± 0.058
	**low Q**				
	Monomers	1.15 ± 0.02	186.1 ± 3.7	2.4 ± 0.1	0.855 ± 0.003
	Oligomers	6.14 ± 1.60	541.5 ± 47.0	3.1 ± 0.1	0.375 ± 0.001
	Fibrils	11.64 ± 0.69	771.2 ± 33.1	2.0 ± 0.1	0.547 ± 0.002
Aβp${}_{3–42}$					
	**high Q**				
	Monomers	0.32 ± 0.01	11.3 ± 0.4	1.0 ± 0.1	0.567 ± 0.011
	Oligomers	0.09 ± 0.01	8.4 ± 0.9	1.3 ± 0.6	0.285 ± 0.014
	Fibrils	0.16 ± 0.02	8.3 ± 0.8	1.3 ± 0.6	0.396 ± 0.022

## Abbreviations

The following abbreviations are used in this manuscript:

AD	Alzheimer’s disease
SANS	Small angle neutron scattering
SDS-PAGE	Sodium dodecyl sulfate–poly-acrylamide gel electrophoresis
